# Meta-omic signatures of microbial metal and nitrogen cycling in marine oxygen minimum zones

**DOI:** 10.3389/fmicb.2015.00998

**Published:** 2015-09-28

**Authors:** Jennifer B. Glass, Cecilia B. Kretz, Sangita Ganesh, Piyush Ranjan, Sherry L. Seston, Kristen N. Buck, William M. Landing, Peter L. Morton, James W. Moffett, Stephen J. Giovannoni, Kevin L. Vergin, Frank J. Stewart

**Affiliations:** ^1^School of Earth and Atmospheric Sciences, Georgia Institute of Technology Atlanta, GA, USA; ^2^School of Biology, Georgia Institute of Technology Atlanta, GA, USA; ^3^Department of Biology, Alverno College Milwaukee, WI, USA; ^4^College of Marine Science, University of South Florida St. Petersburg, FL, USA; ^5^Department of Earth, Ocean and Atmospheric Sciences, Florida State University Tallahassee, FL, USA; ^6^Department of Biological Sciences, University of Southern California Los Angeles, CA, USA; ^7^Department of Microbiology, Oregon State University Corvallis, OR, USA

**Keywords:** oxygen minimum zones, metalloenzymes, iron, copper, denitrification, anammox, metagenomes, metatranscriptomes

## Abstract

Iron (Fe) and copper (Cu) are essential cofactors for microbial metalloenzymes, but little is known about the metalloenyzme inventory of anaerobic marine microbial communities despite their importance to the nitrogen cycle. We compared dissolved O_2_, NO${}_{3}^{-}$, NO${}_{2}^{-}$, Fe and Cu concentrations with nucleic acid sequences encoding Fe and Cu-binding proteins in 21 metagenomes and 9 metatranscriptomes from Eastern Tropical North and South Pacific oxygen minimum zones and 7 metagenomes from the Bermuda Atlantic Time-series Station. Dissolved Fe concentrations increased sharply at upper oxic-anoxic transition zones, with the highest Fe:Cu molar ratio (1.8) occurring at the anoxic core of the Eastern Tropical North Pacific oxygen minimum zone and matching the predicted maximum ratio based on data from diverse ocean sites. The relative abundance of genes encoding Fe-binding proteins was negatively correlated with O_2_, driven by significant increases in genes encoding Fe-proteins involved in dissimilatory nitrogen metabolisms under anoxia. Transcripts encoding cytochrome c oxidase, the Fe- and Cu-containing terminal reductase in aerobic respiration, were positively correlated with O_2_ content. A comparison of the taxonomy of genes encoding Fe- and Cu-binding vs. bulk proteins in OMZs revealed that Planctomycetes represented a higher percentage of Fe genes while Thaumarchaeota represented a higher percentage of Cu genes, particularly at oxyclines. These results are broadly consistent with higher relative abundance of genes encoding Fe-proteins in the genome of a marine planctomycete vs. higher relative abundance of genes encoding Cu-proteins in the genome of a marine thaumarchaeote. These findings highlight the importance of metalloenzymes for microbial processes in oxygen minimum zones and suggest preferential Cu use in oxic habitats with Cu > Fe vs. preferential Fe use in anoxic niches with Fe > Cu.

## Introduction

Marine oxygen minimum zones (OMZs) play important roles in global biogeochemical cycles and are expanding throughout the world's oceans (Stramma et al., 2008; Keeling et al., 2010). OMZs occur where respiration of O_2_ exceeds resupply, which in turn draws down O_2_ concentrations potentially to anoxia. Nitrogen (N) cycling has been a particular focus in OMZ research due to the major contribution of these regions to global fixed N loss to the atmosphere. The N cycle in OMZs is dominated by nitrate reduction to N_2_ (denitrification) and anaerobic ammonia oxidation (anammox) and in some cases also dissimilatory nitrate reduction to ammonium (DNRA; Lam and Kuypers, 2011), while nitrification has been shown to be an important source of oxidized N at OMZ boundaries (Ward and Zafiriou, 1988; Ward et al., 1989; Lipschultz et al., 1990; Füssel et al., 2011). Diverse organisms mediate OMZ N cycling, but members of the Planctomycetes, Thaumarchaeota and Nitrospinae phyla appear to perform the majority of anammox, ammonia oxidation and nitrite oxidation, respectively, based on rate measurements coupled to primer-based 16S rRNA and functional gene sequencing, as well as metagenomic and metatranscriptomic approaches (Lam et al., 2009; Füssel et al., 2011; Newell et al., 2011; Stewart et al., 2012; Ulloa et al., 2012; Wright et al., 2012; Ganesh et al., 2014, 2015; Hawley et al., 2014).

Numerous enzymes involved in N cycling, photosynthesis, and respiration require metal cofactors, with the two most important redox-active metals being Fe and Cu (Morel and Price, 2003; Godfrey and Glass, 2011; Glass and Orphan, 2012). Copper is a potentially limiting micronutrient for marine ammonia-oxidizing Thaumarchaeota (Walker et al., 2010; Amin et al., 2013; Jacquot et al., 2014) and diverse denitrifying bacteria that use Cu-containing metalloenzymes for nitrite and nitrous oxide reduction (Granger and Ward, 2003; Twining et al., 2007; Pomowski et al., 2011; Felgate et al., 2012). While Fe has been shown to limit N_2_ fixation and photosynthesis in surface oxic seawater (Sohm et al., 2011; Morrissey and Bowler, 2012), the distribution and expression of genes encoding microbial metalloproteins at the community level in marine OMZs is not well characterized. A recent study showed that Fe availability might be important for supporting N_2_ fixation in OMZs (Loescher et al., 2014). Other studies have investigated relationships between Cu availability and rates of denitrification and ammonia oxidation (Ward et al., 2008; Jacquot et al., 2014). In pure culture studies, Fe has also been shown to be an important micronutrient, electron acceptor (as Fe^3+^) and electron donor (as Fe^2+^) for anammox bacteria (Van De Vossenberg et al., 2008; Oshiki et al., 2013; Van de Vossenberg et al., 2013; Ali et al., 2014), suggesting that it may play a role in marine OMZs where anammox occurs.

Total dissolved Cu concentrations tend to be higher (0.5–5 nM; Bruland and Franks, 1983) than Fe (0.05–0.7 nM; Johnson et al., 1997) in the open ocean, particularly in ocean regions far from coasts with minimal inputs of dust (Boyd and Ellwood, 2010; Moore et al., 2013). Higher availability of Cu vs. Fe in the open ocean may provide a selective advantage for some phytoplankton that can substitute functionally equivalent Cu-containing enzymes in place of Fe-binding proteins for photosynthesis (Peers and Price, 2006). In contrast to oxic, slightly alkaline (pH ~8.2) seawater, OMZs stabilize reduced Fe^2+^ due to their lower pH (~7.5; Paulmier and Ruiz-Pino, 2009) and O_2_ content (Hopkinson and Barbeau, 2007; Moffett et al., 2007; Kondo and Moffett, 2013, 2015; Vedamati et al., 2014). Steep gradients in metal concentrations have been observed in OMZs (Hopkinson and Barbeau, 2007; Moffett et al., 2007; Jacquot et al., 2013; Vedamati et al., 2014; Kondo and Moffett, 2015), suggesting that these regions may be ideal features for exploring linkages between metal availability and microbial community gene content and expression.

We hypothesized that the O_2_ content of seawater and the molar ratio of total dissolved Fe to Cu may affect the relative percentage of functional genes and transcripts encoding Fe-utilizing proteins vs. Cu-utilizing proteins in marine microbial communities. We tested the hypothesis by coupling trace metal profiles and meta-omic datasets spanning oxic to anoxic gradients in the Eastern Tropical North Pacific (ETNP) off Manzanillo, Mexico and the Eastern Tropical South Pacific (ETSP) off Iquique, Chile, in comparison to a fully oxic “control” profile from the Bermuda Atlanta Time-series Station (BATS) in the North Atlantic Sargasso Sea, as well as basin-scale transects of O_2_ and trace metal concentrations in the Atlantic and Pacific oceans (Supplementary Figure 1). In the ETNP and ETSP OMZs and at BATS, O_2_, NO${}_{3}^{-}$, NO${}_{2}^{-}$, Fe and Cu concentrations were analyzed relative to the proportional abundances and taxonomic identities of genes encoding catalytic proteins using a custom database containing 108 Fe- and 16 Cu-binding protein fold families (Dupont et al., 2006, 2010). Twenty-eight metagenomes were analyzed: twelve from stations 1 and 3 in the ETSP (15–1000 m depth), nine from stations 6 and 10 in the ETNP (30–300 m depth) and seven from BATS (1–250 m depth). In addition, nine metatranscriptomes were analyzed: five from station 6 in the ETNP (30–300 m depth) and four from station 3 in the ETSP (50–200 m depth), with particular emphasis on Fe- and Cu-metalloenzymes involved in O_2_ and N cycling. Trends at the community level were then compared to the abundances of genes encoding Fe- and Cu-metalloenzymes in genomes of Planctomycetes, Thaumarchaeota and Nitrospinae species closely related to those that drive anammox, ammonia oxidation and nitrite oxidation, respectively, in OMZs: *Scalindua profunda*, an anammox planctomycete most active in OMZ cores (Kuypers et al., 2003, 2005; Schmid et al., 2007; Woebken et al., 2008; Galan et al., 2009; Lam et al., 2009), *Nitrosopumilus maritimus*, an aerobic ammonia-oxidizing thaumarchaeote most active in the oxycline and uppermost OMZ (Francis et al., 2005; Lam et al., 2007; Beman et al., 2012; Stewart et al., 2012), and *Nitrospina gracilis*, an aerobic nitrite-oxidizing bacterium most active in the upper OMZ and into the core OMZ (Füssel et al., 2011; Beman et al., 2013).

## Materials and methods

### Sample collection and geochemical analysis

In the ETNP, samples were collected aboard the R/V *New Horizon* cruise NH-1315 in June 2013 offshore Manzanillo, Mexico at station 2 (18.9°N, 108.8°W), station 4 (18.9°N, 106.3°W), station 6 (18.9°N, 104.5°W) and station 10 (18.8°N, 104.7°W) at depths ranging from 30 to 500 m (Supplementary Figure 1). Seawater for meta-omic analysis and N geochemistry was sampled from five depths (30, 85, 100, 125, and 300 m) at station 6 and four depths (30, 80, 125, and 300 m) at station 10 using Niskin bottles deployed on a rosette containing a CTD profiler (Sea-Bird SBE 911plus) equipped with a dissolved O_2_ (SBE43) sensor. Microbial biomass (0.2–1.6 μm) was collected on Sterivex filters as described by Ganesh et al. (2014) for DNA and Ganesh et al. (2015) for RNA. The time interval between sample capture and final preservation of RNA samples was generally < 30 min and no more than 45 min. Nitrate and nitrite concentrations were determined using chemiluminescence after reduction to nitric oxide with acidic vanadium (III) (Braman and Hendrix, 1989). Nitrite was determined spectrophotometrically through a modified Griess reaction (Grasshoff, 1983) on fresh samples collected from the Niskin bottles. Nitrite concentrations were subsequently subtracted to obtain NO${}_{3}^{-}$ concentrations.

At the BATS site (31.67°N, 64.17°W), samples from seven depths (1, 40, 80, 120, 160, 200, and 250 m) were collected in August 2002 on cruise BATS-167. Environmental parameters (O_2_, NO${}_{2}^{-}$ and NO${}_{3}^{-}$) were measured following standard BATS methods (Knap et al., 1997). Microbial biomass was collected on 0.2-μm polyethersulfone membranes, and nucleic acids were extracted and purified as described previously (Giovannoni et al., 1996; Morris et al., 2005; Treusch et al., 2009).

Samples for total dissolved iron (dFe_T_) and copper (dCu_T_) analyses were taken using GO-FLO (General Oceanics) or Niskin-X (Ocean Test Equipment) trace metal clean bottles on a plastic-coated hydrowire on ETNP cruise NH-1315 in 2013, ETSP cruise AT-15-61 in 2010 [station 10 (10°S, 86°W; dFe_T_ profile previously reported in Kondo and Moffett, 2015) and station 11 (10°S, 82.5°W; dFe_T_ and dCu_T_ profiles previously reported in Jacquot et al., 2013; Kondo and Moffett, 2015), respectively], CLIVAR cruise A16N in 2003 (Atlantic transect from 62°N to 5°S along 20 to 30°W; dFe_T_ profiles previously reported in Measures et al., 2008), CLIVAR cruises P16N in 2005 and P16S in 2006 (Pacific transect from 37°N to 46°S along 150°W) and GEOTRACES inter-comparison cruise at BATS (31.8°N, 64.1°W) in 2008 (dFe_T_ and dCu_T_ profiles previously reported in Milne et al., 2010). After sampling, seawater was filtered through an Acropak-200 capsule into trace metal clean LDPE bottles according to established GEOTRACES protocols (Cutter et al., 2014). Each sample was acidified to 0.024 M HCl with trace metal grade HCl (BDH Arista Ultra) and stored double-bagged until analysis on an ELEMENT 2 magnetic sector HR-ICP-MS using an established protocol (Milne et al., 2010). Samples for metal speciation were taken as described above, except 1 L of water was filtered into trace metal clean FLPE bottles, which were subsequently frozen at −20°C. The organic complexation of dissolved Fe and Cu was quantified using competitive ligand exchange-adsorptive cathodic stripping voltammetry (CLE-ACSV) with the added ligand salicylaldoxime (SA) as described in Buck et al. (2012).

### DNA and RNA extraction, meta-omic sequencing and bioinformatic analysis

DNA was extracted from Sterivex filters using a phenol:chloroform protocol as described in Ganesh et al. (2014). RNA was extracted from Sterivex filters using a modification of mirVana™ miRNA Isolation Kit (Ambion) as described in Ganesh et al. (2015). For ETNP samples, Illumina sequencing on a MiSeq platform was used to characterize the community DNA (metagenome) and RNA (metatranscriptome) from purified DNA and RNA, respectively, from the Sterivex filter fraction (0.2–1.6 μm). Barcoded sequencing libraries were prepared with Nextera XT technology (Illumina) and used for paired end (2X250 bp) sequencing on two MiSeq runs (one for each station). Sequences from the Sterivex size fraction (0.2–1.6 μm) from the ETSP were generated in prior studies as described previously for MOOMZ/station 3 (Stewart et al., 2012) and BIGRAPA/station 1 (Ganesh et al., 2014). For the BATS samples, metagenomic library construction and shotgun sequencing were performed using the 454 Life Sciences standard GS FLX protocol and a Roche GS FLX sequencer (454 Life Sciences).

Sequence statistics for ETNP and BATS metagenomes reported here for the first time are provided in Supplementary Table 1. ETNP metagenomic sequences are publically available under NCBI BioProject ID PRJNA254808 (BioSample accession numbers SAMN02905556-SAMN02905564) and Sequence Read Archive Project ID SRP044185. BATS metagenomic sequences are available on iMicrobe under Project Code CAM_PROJ_BATS (BATS_SMPL_BATS-167-0 through BATS_SMPL_BATS-167-0250). Sequence statistics for previously published metatranscriptomes (ETSP MOOMZ/station 3 and ETNP station 6) and ETSP metagenomes (MOOMZ/station 3 and BIGRAPA/station 1) can be found in Stewart et al. (2012) and Ganesh et al. (2014, 2015). Previously published sequences are available under the following NCBI BioProject/SRA Project IDs: PRJNA68419/SRP003331 (ETSP metatranscriptomes), PRJNA217777/SRP029388 (ETSP metagenomes), and PRJNA263621/SRP052876 (ETNP metatranscriptomes).

Analysis of protein-coding metagenomic and metatranscriptomic sequences followed that of Stewart et al. (2012) and Ganesh et al. (2014, 2015). Illumina reads were filtered by quality (Phred score 25) and high-quality paired reads were merged using custom scripts incorporating the FASTX toolkit (http://hannonlab.cshl.edu/fastx_toolkit/index.html). Merged sequences were queried using BLASTX against the NCBI-nr database of non-redundant protein sequences as of November 2013. BLASTX matches above a bit-score of 50 were retained and used for further analysis. Gene/transcript identities were determined from the top reference gene(s) matching each query read via BLASTX (above a bit score cutoff of 50). For reads matching multiple reference genes with equal bit score, each matching reference was retained as a top hit, with its representation scaled proportionately to the number of genes sharing an equal bit score. The 454 sequences were processed in the same manner as Illumina reads, except they were not merged because they were not paired-end reads. The taxonomic composition of protein coding sequences was determined based on the taxonomic annotation of each gene according to the NCBI-nr taxonomy in MEGAN5 (Huson et al., 2011; min score: 50; max expected: 0.01; top percent 10; min complexity: 0.3).

The relative abundance of genes encoding Fe and Cu-binding proteins for the ETNP, ETSP and BATS metagenomes, and three published genomes (*Scalindua profunda*, JGI 2017108002/2022004002 (Van de Vossenberg et al., 2013); *Nitrosopumilus maritimus*, NCBI accession NC_010085.1 (Walker et al., 2010); *Nitrospina gracilis*, NZ_CAQJ00000000.1/EMBL-EBI project number PRJEB1269 Lücker et al., 2013) was estimated via BLASTX (e-value 0.1, bitscore 50) versus a custom database of all Fe and Cu-binding protein fold families (Dupont et al., 2006, 2010) from the extended Structural Classification of Proteins database (SCOPe; Fox et al., 2014; October 2013 version; http://scop.berkeley.edu). The SCOPe database contains a hierarchy of protein families and sequence(s) associated with each protein, each with a unique SCOPe identifier, the “sunID.” We obtained the sunID for each family of interest from Dupont et al. (2006, 2010), and then recursively traversed the SCOPe hierarchy to produce a list of sunIDs for all proteins within that family. We then iterated over each list of protein sunIDs, retrieved the sequence data, and appended it to a fasta file to produce one reference file for Cu and Fe protein families (available for download at http://www.glass.eas.gatech.edu/wp-content/uploads/2014/08/cu_fam.zip and http://www.glass.eas.gatech.edu/wp-content/uploads/2014/08/fe_fam.zip). Fold families representing < 4% of total hits were clustered together as “others.” By matching genes in the multidomain cupredoxins (SCOPe b.6.1.3) to the BLAST output from the nr database and extracting the gene description, we divided the SCOPe b.6.1.3 family into nitrite reductase/*nirK* and multicopper oxidases (MCOs). SCOPe b.6.1.3 sequences not belonging to either *nirK* or MCOs were added as a percentage of “others.” The Fe regulatory protein aconitase (SCOPe c.83.1.1) was removed to limit the output to catalytic Fe proteins. Phylum-level taxonomic profiles of gene sequences encoding Fe and Cu-binding proteins were obtained using MEGAN5 (Huson et al., 2011; min score: 50; max expected: 0.01; top percent 10; min complexity: 0.3). Relative gene abundances were normalized to the total number of protein-coding genes (Supplementary Table 1) from the nr BLAST multiplied by 100,000.

Statistical analysis was performed using Spearman's rank correlations with R software. The variance contribution of environmental factors on Fe and Cu gene abundances based on their OMZ zone was determined using a partial canonical correspondence analysis (CCA; “vegan” package in R, Oksanen et al., 2002). Depth, temperature, O_2_, NO${}_{3}^{-}$, NO${}_{2}^{-}$, PO${}_{4}^{3-}$, Fe, and Cu concentrations from the ETNP 2013 cruise metadata were used as environmental variables. Prior to running the CCA, relative abundances of genes and transcripts from metagenomes and metatranscriptomes, respectively, were normalized using the rarefaction function in R (rarefy; "vegan" package in R, 999 permutations). The effect of environmental variables was visualized using ordination plots in R.

## Results and discussion

### Influence of oxygen on nitrogen and metal geochemistry

**Figure 1:**
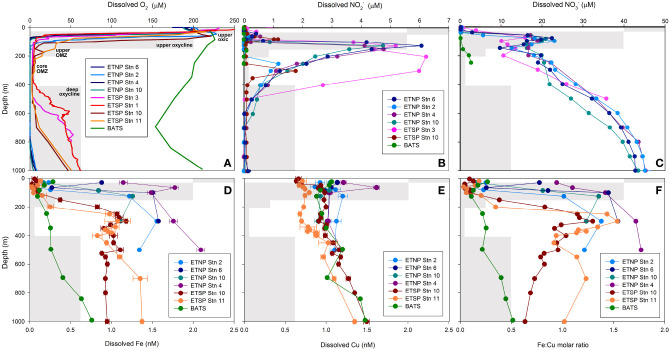
**Depth profiles of dissolved (A) O_2_, (B) NO${}_{2}^{-}$, (C) NO${}_{3}^{-}$, (D) Fe, (E) Cu and (F) Fe:Cu molar ratios for stations 2, 4, 6 and 10 in the ETNP, stations 1 (BIG RAPA) and 3 (MOOMZ), 10 and 11 in the ETSP, and BATS in the Sargasso Sea, North Atlantic Ocean (see Supplementary Figure 1 for station maps)**. Gray boxes depict oxygen and depth ranges for each zone and their labels are shown in **(A)**.

Oxygen and depth profiles were used to identify five zones through ETNP and ETSP OMZs: the upper oxic zone (15–30 m; >200 μM O_2_), the upper oxycline (50–85 m; 10–200 μM O_2_), the upper OMZ (70–125 m; < 10 μM O_2_), the core OMZ (200–300 m; < 5 μM O_2_) and the lower oxycline (500–1000 m; 5–50 μM O_2_). The base of the upper oxycline deepened with increasing distance from shore, from 70 m at ETNP station 6, to 200 m at ETSP stations 10 and 11 (Figure 1A, Supplementary Figure 1). The lower oxycline was deeper in the ETNP (800–900 m) than in the ETSP (400–700 m; Figure 1A). The secondary NO${}_{2}^{-}$ maximum was most pronounced at nearshore stations (5–6 μM NO${}_{2}^{-}$ at ETNP stations 4, 6, and 10 at ETSP station 3) and was weaker and deeper at offshore stations (1–2 μM NO${}_{2}^{-}$ at ETNP station 2 and ETSP station 10; Figure 1B). Secondary NO${}_{2}^{-}$ maxima corresponded with NO${}_{3}^{-}$ minima in all OMZ stations (Figure 1C). At BATS, O_2_ did not drop below 150 μM and NO${}_{2}^{-}$ was undetectable at all depths, while NO${}_{3}^{-}$ increased from under detection at 0–120 m to 3 μM at 250 m (Figures 1A–C).

Total dissolved Fe (dFe_T_) and labile inorganic Fe complexes [Fe′] concentrations increased with depth at all stations (Figure 1D; Supplementary Table 2). Depth gradients were steeper and dFe_T_ reached higher maxima in OMZs (1–2 nM) than at BATS (0.8 nM; Figure 1D). In general, dFe_T_ and [Fe'] were elevated closer to shore (e.g., at ETNP stations 4, 6, and 10 vs. ETNP station 2; Supplementary Table 2; Figure 1D). In contrast, dCu_T_ (0.9–1.6 nM) and log Cu^2+^ (−13.9 to −15 M) concentrations did not vary significantly with O_2_ content or distance from shore (Figure 1E; Supplementary Table 3). These trends are consistent with previous studies showing strong increases in dFe_T_ in the upper OMZs in the ETNP, ETSP, and Arabian Sea (Hopkinson and Barbeau, 2007; Moffett et al., 2007; Vedamati et al., 2014; Kondo and Moffett, 2015) and more gradual increases in dCu_T_ with depth in the ETSP OMZ (Jacquot et al., 2013).

The “L_1_” ligand class notation is used to designate the strongest Fe and Cu-binding ligands with the highest conditional stability constants (log$K_{FeL,Fe^{\prime }}^{cond}$ > 12; Gledhill and Buck, 2012). In the ETNP OMZ, concentrations of Fe- and Cu-binding L_1_ligands were consistently in excess of total dissolved metal concentrations (1.1–2.9 nM L_1_ Fe; 2.0–5.8 nM L_1_Cu), and conditional stability constants were elevated at all stations and depths (12.4–12.9 log $K_{FeL,Fe^{\prime }}^{cond}$; 13.8–14.8 log $K_{CuL,Cu^{2+}}^{cond}$; Supplementary Tables 2, 3), suggesting that nearly all Fe^3+^ and Cu^2+^ would be bound to these strong organic ligands at equilibrium, as previously reported for Fe in the ETNP (Hopkinson and Barbeau, 2007) and both Fe and Cu in the ETSP (Jacquot et al., 2013; Kondo and Moffett, 2015).

Ratios of dFeT:dCuT (hereafter referred to as Fe:Cu molar ratios) generally followed the same trends as dFe_T_ because dCu_T_ increased only slightly between the upper oxic zone and the upper/core OMZ. Fe:Cu molar ratios >1 were only found in upper/core OMZ and lower oxycline samples, whereas ratios in upper oxic and oxycline waters ranged from 0.03 to 0.9 (Figure 1F). At BATS, Fe:Cu molar ratios were consistently < 1 and increased with depth to a maximum of 0.6 at 1000 m (0.1–0.8 nM dFe_T_; 0.8–2.2 nM dCu_T_; Figures 1D–F), consistent with the release of Fe due to remineralization of sinking particles as observed throughout the world's oceans (Boyd and Ellwood, 2010).

### Fe:Cu ratios in atlantic vs. pacific transects

**Figure 2:**
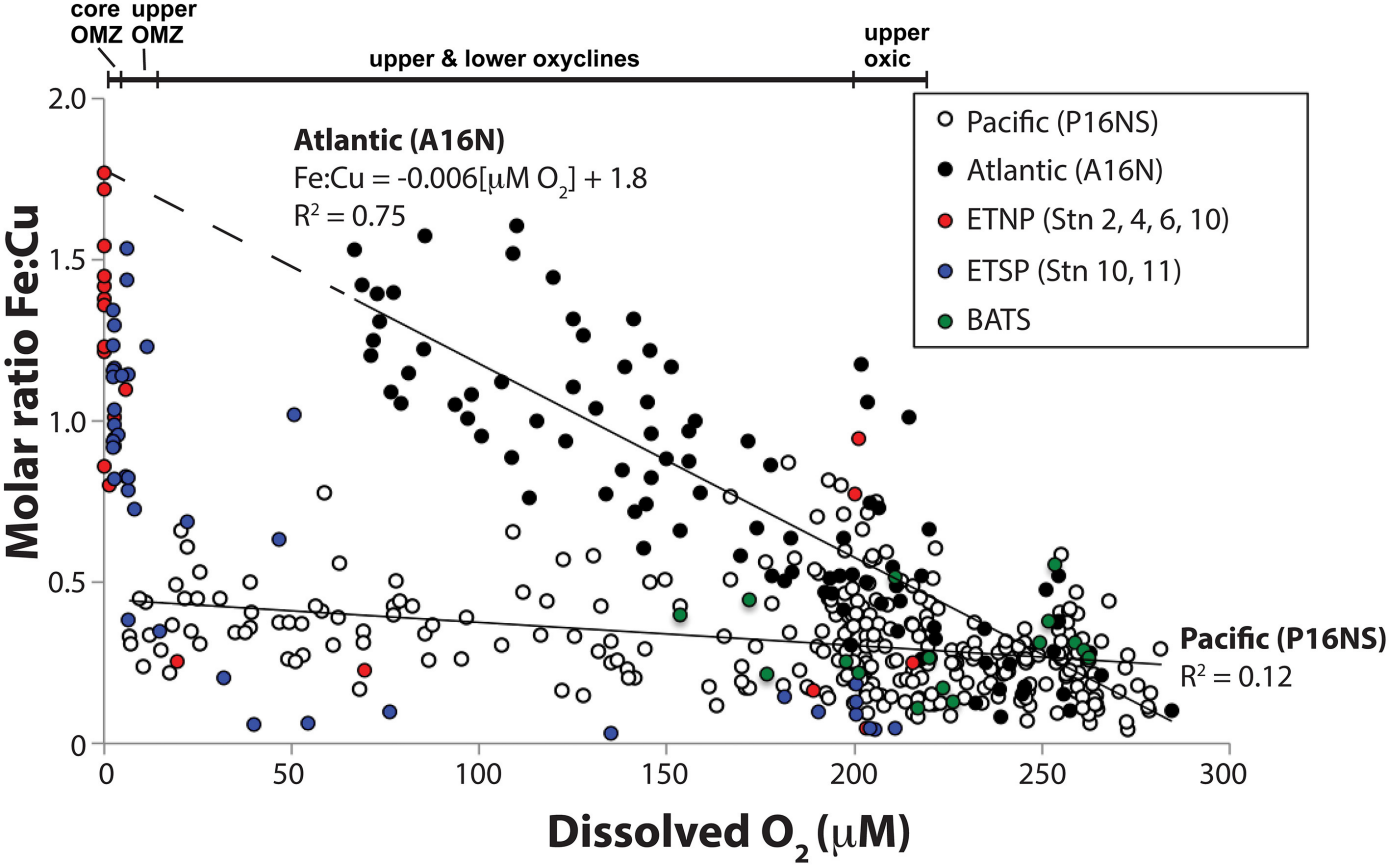
**Relationship between total dissolved Fe:Cu molar ratios and dissolved O_2_ concentrations in seawater samples from 0 to 1000 m water depth**. Closed circles are Atlantic Ocean samples taken along the A16N transect from 62°N to 5°S along 20–30°W on CLIVAR cruise A16N (see Supplementary Figure 1 for station maps). Open circles are Pacific Ocean samples taken along the CLIVAR cruise P16N and P16S transect from 37°N to 46°S along 150°W (Supplementary Figure 1). Red, blue and green circles ETNP NH-1315, ETSP AT-15-61, and BATS samples, respectively, for which full depth profiles are shown in Figure 1F. A linear fit equation of the Atlantic Ocean data (*R*^2^ = 0.75) is provided. A linear fit equation is not provided for the Pacific Ocean data due to the low *R*^2^ value (0.12).

Molar Fe:Cu ratios vs. O_2_ were compared on an ocean basin scale to identify global gradients over which metalloenzyme distributions were predicted to vary. Fe:Cu ratios for seawater samples from 0 to 1000 m water depth were compiled from Atlantic and Pacific CLIVAR ocean basin scale cruises (Figure 2). Fe:Cu molar ratios from the Atlantic A16N transect ranged from 0.08 to 1.6 (0.08–1.5 nM dFe_T_; 0.5–1.3 nM dCu_T_) over 67–284 μM O_2_ with a significant inverse relationship between Fe:Cu ratios and O_2_ concentrations that could be fit to the linear equation Fe:Cu molar ratio = −0.006[μM O_2_] + 1.8 (*R*^2^ = 0.75; Figure 2). Highest Fe:Cu and lowest O_2_ along the Atlantic transect were found at 200–600 m depth in the tropics (~4–14°N).

Fe:Cu molar ratios from the Pacific Ocean transect (P16NS) ranged from 0.03 to 0.9 (0.03–1.9 nM dFe_T_; 0.4–2.8 nM dCu_T_). Lowest O_2_ concentrations (7 μM) in the Pacific transect occurred at 1000 m in the subtropical North Pacific. The strong inverse relationship between Fe:Cu and O_2_ in the Atlantic CLIVAR transect was not observed in the Pacific P16NS transect (*R*^2^ = 0.12). The difference in the strength of the response of Fe:Cu to O_2_ may be a function of Fe supply in the Atlantic vs. Pacific. Saharan dust inputs to the equatorial North Atlantic cause persistent elevated Fe concentrations throughout the upper 1000 m as shown by Measures et al. (2008), whereas dust supply is lower and Fe inputs are limited to upwelling, remineralization from settling organic debris, and lateral mixing from the reducing shelf and slope sediments in the Pacific (Hopkinson and Barbeau, 2007).

Seawater samples from ETNP and ETSP OMZs with < 7 μM O_2_ possessed higher Fe:Cu ratios (0.8–1.8) than all CLIVAR and BATS samples. Two outliers with elevated Fe:Cu ratios (0.8–0.9) at ~200 μM O_2_ were nearshore ETNP samples potentially influenced by significant Fe input from rivers or an upwelled sediment source. Interestingly, the predicted maximum Fe:Cu ratio based on the linear fit equation of the Atlantic data was identical to the highest Fe:Cu ratio measured in the core of the ETNP OMZ (Fe:Cu = 1.8; Figures 1F, 2), potentially suggesting similar limits on Fe remobilization at trace O_2_ levels and a potential upper bound for Fe:Cu ratios in the pelagic zone.

### Taxonomic composition of bulk protein coding vs. Cu and Fe genes and transcripts

**Figure 3:**
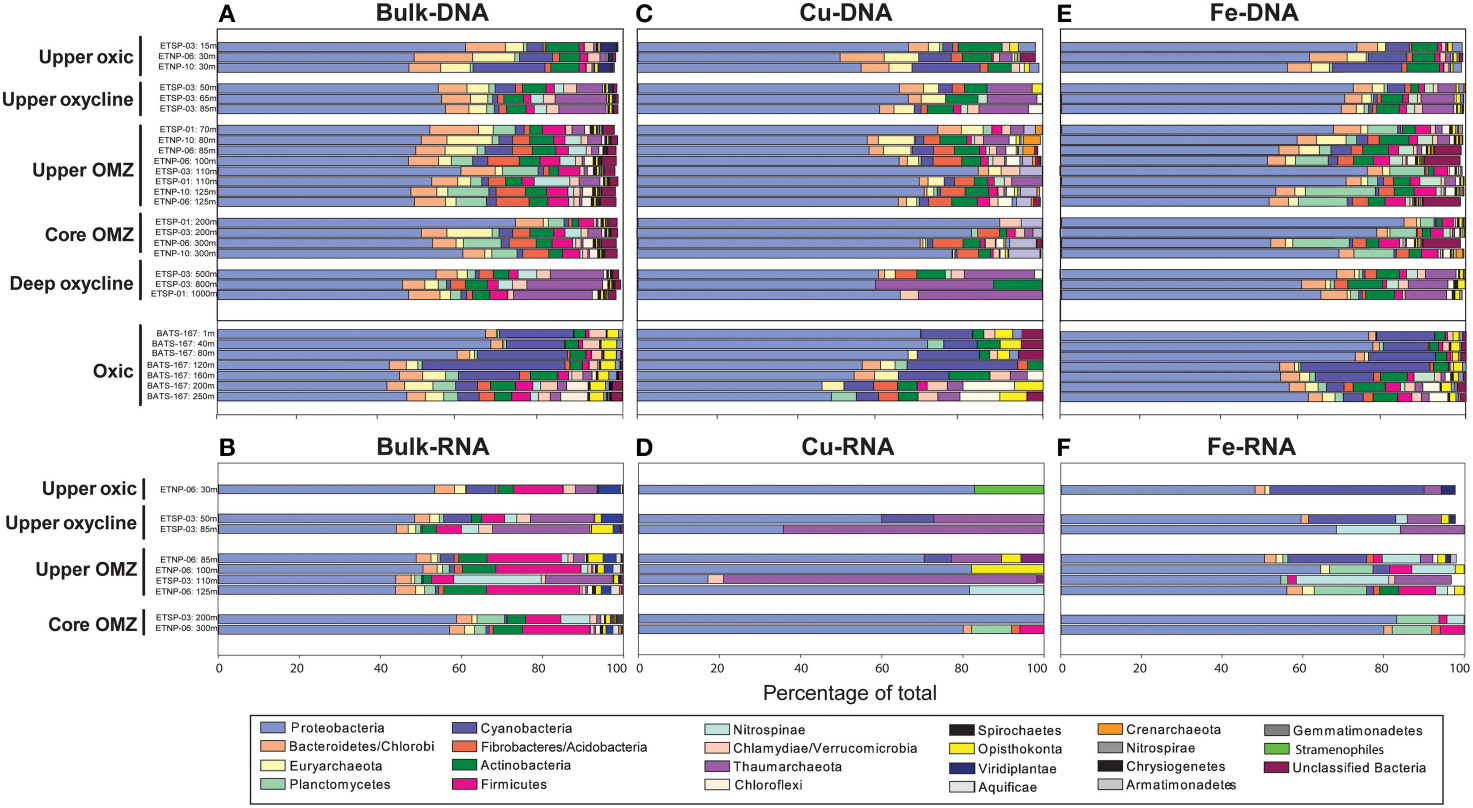
**Taxonomy of metagenomic (A,C,E) and metatranscriptomic (B,D,F) sequences for bulk protein-coding genes (A,B), Cu-binding proteins (C,D), and Fe-binding proteins (E,F) through five zones in ETNP and ETSP OMZs and at BATS**. The OP1 candidate phylum is not listed because it was not included in the MEGAN database, but comprises the majority of formate dehydrogenase transcripts classified as dissimilatory membrane-bound nitrate reductase in OMZ cores based on BLASTX analysis.

Bacteria comprised 75–96% of total protein-coding DNA and RNA sequences in OMZ and BATS datasets (Supplementary Table 4). At the phylum level, Proteobacteria were consistently dominant (42–63% of total DNA and RNA protein-coding sequences; Figures 3A,B). Of the known N-cycling phyla, Planctomycetes (including anammox-capable Brocadiales) reached 10% of total DNA and RNA sequences within OMZs and Nitrospinae comprised up to 20% of total RNA sequences in the upper OMZ in the ETSP. Archaea comprised 0–9% of total protein-coding DNA sequences at BATS vs. 2–22% of total DNA and RNA protein-coding sequences in OMZs (Supplementary Table 4), with notable peaks of Thaumarchaeota in the oxyclines and upper OMZ (Figures 3A,B). Eukaryotes, dominated by Opisthokonta, Stramenopiles and Viridiplantae, comprised 1–5% of total protein-coding DNA sequences in OMZs vs. 6–8% at BATS (Figure 3A; Supplementary Table 4). The slightly higher percentage of eukaryotes at BATS may be due to the inclusion of larger cells in BATS samples where a pre-filter was not used and thus all cells >0.2 μm were collected vs. OMZ metagenomes comprised solely of the 0.2–1.6 μm size fraction. Alternatively, microbial eukaryotes may be relatively more abundant in oxic waters.

The taxonomy of genes encoding Cu and Fe proteins was broadly similar to that of the bulk taxonomy, except Planctomycetes were an insignificant contributor to Cu genes (Figure 3C) and represented a higher percentage of Fe genes in OMZs (up to 18%; Figure 3E). Conversely, Thaumarchaeota represented a higher percentage of Cu genes, particularly in lower oxyclines underlying ETSP OMZs (up to 30%; Figure 3C) and a lower percentage of Fe genes (Figure 3E).

The taxonomy of Cu and Fe transcripts showed notable differences when compared to bulk transcripts or Cu and Fe genes from metagenomes. First, the phylogenetic diversity of Cu and Fe transcripts at the phylum-level was notably lower than Cu and Fe genes; Cu transcripts were associated with only 1-5 phyla (Figure 3D) and Fe transcripts were associated with only 3-13 phyla (Figure 3F). Second, Cu transcripts were highly enriched in Thaumarchaeota sequences (up to 77% of total Cu transcripts in ETSP station 3 at 110 m; Figure 3D). Third, Fe transcripts showed a higher percentage of Cyanobacteria in the upper oxic zone compared to bulk transcripts and Fe genes (up to 38%; Figure 3F).

### Copper genes and transcripts: relative abundance and functional composition

**Figure 4:**
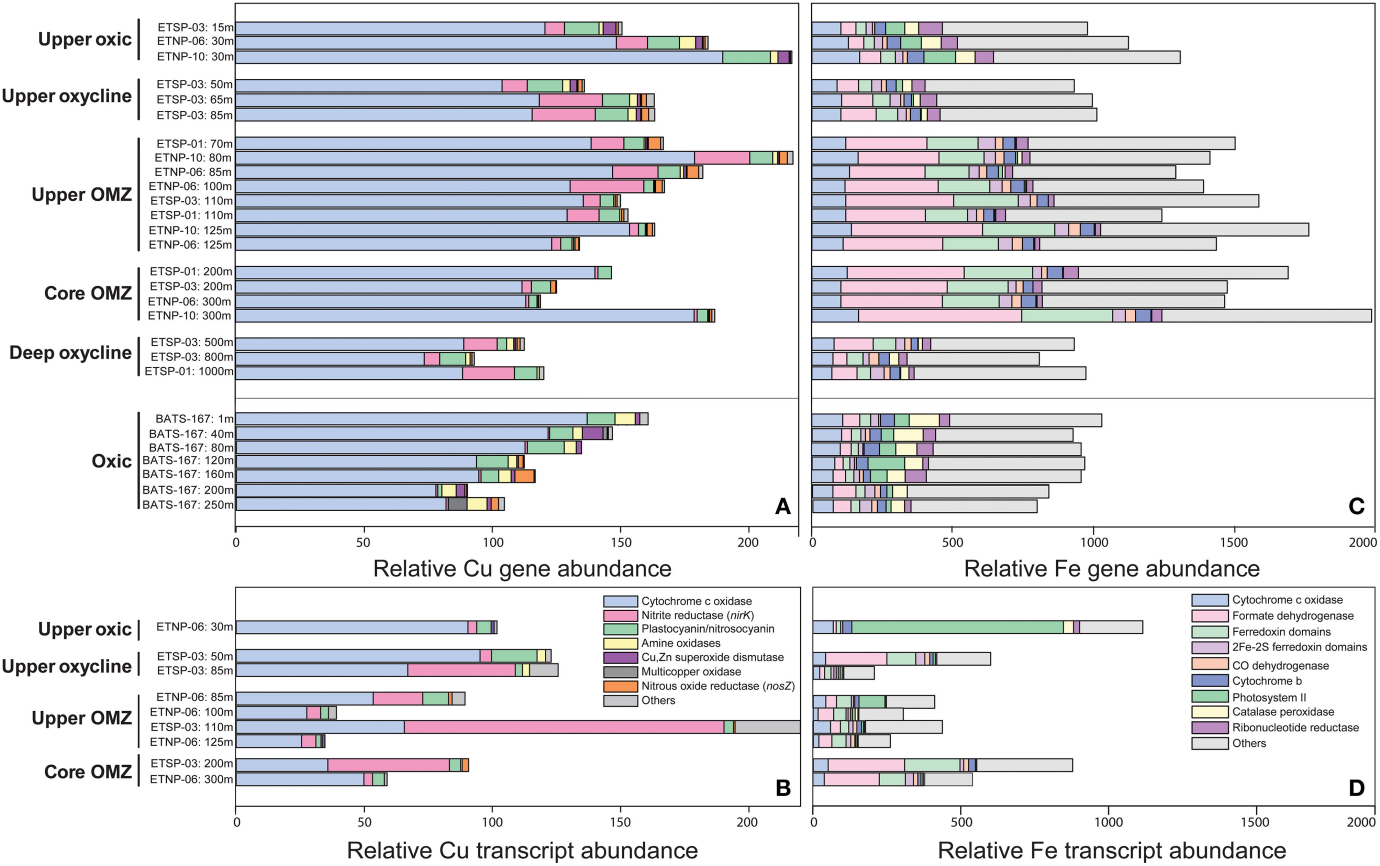
**Relative abundance of sequences from ETNP, ETSP, and BATS metagenomes (A,C) and ETNP and ETSP metatranscriptomes (B,D) normalized to total protein-coding genes or transcripts and multiplied by 100,000**. Genes shown encode **(A,B)** Cu-binding proteins [SCOPe ID: cytochrome c oxidase subunit I-like (f.24.1.1) and subunit-II-like (b.6.1.2), nitrite reductase/NirK (b.6.1.3), plastocyanin/nitrosocyanin (b.6.1.1 and b.6.1.4), amine oxidases and galactose oxidases (b.30.2.1 and b.69.1.1), Cu/Zn superoxide dismutase (b.1.8.1) and nitrous oxide reductase/NosZ (b.69.3.1) and others (b.69.1.5, b.86.1.1, b86.1.2, d.230.3.1, b.6.1.6, b.6.1.7, g.46.1.1)] and **(C,D)** Fe-binding proteins [SCOPe ID: cytochrome c oxidase subunit I-like (f.24.1.1), formate dehydrogenase/DMSO reductase domains 1-3 (c.81.1.1), ferredoxin domains from multidomain families (d.58.1.5), 2Fe-2S ferredoxin domains (d.15.4.2), CO dehydrogenase ISP C-domain-like (a.56.1.1), cytochrome b of cytochrome bc1 complex (ubiquinol-cytochrome c reductase) (f.21.1.2), photosystem II (f.26.1.1), catalase-peroxidase KatG (a.93.1.3), ribonucleotide reductase-like (a.25.1.2) and others (see Supplementary Table 5)].

**Figure 5:**
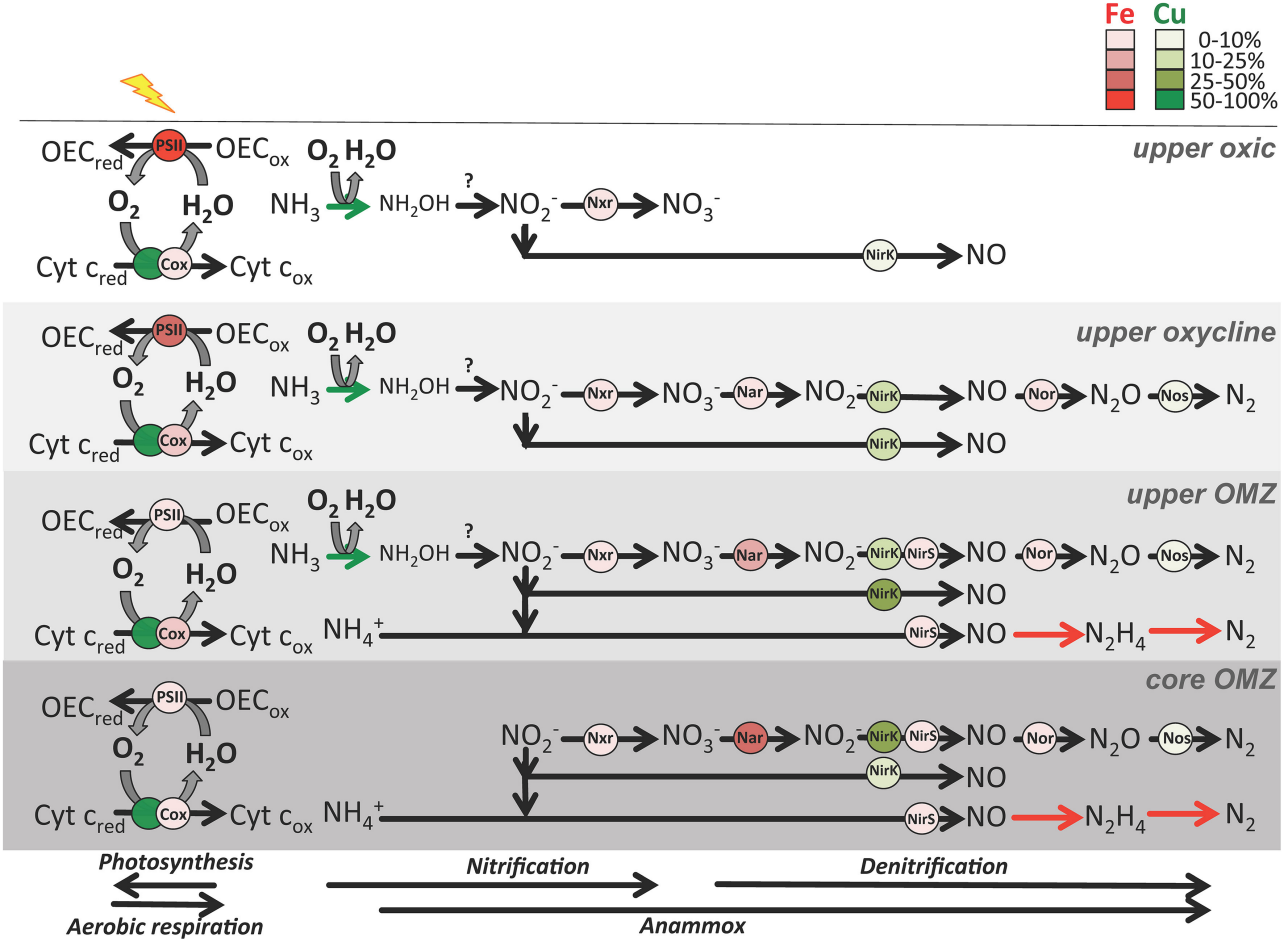
**Schematic of major metabolisms involved in O_2_ (photosynthesis and aerobic respiration) and nitrogen cycling (nitrification, canonical denitrification and anammox) in four zones in OMZs**. Metalloenzymes are depicted as red (Fe) or green (Cu) circles. Shading indicates relative abundance of transcripts normalized to total Fe and Cu transcripts (see legend) from ETNP station 6 and ETSP station 3 metatranscriptomes (see Supplementary Table 5 for transcript abundances). Abbreviations: Cyt c, cytochrome c; OEC, oxygen evolving complex; red, reduced; ox, oxidized; Cox, cytochrome c oxidase; Nar, dissimilatory nitrate reductase; Nxr, nitrite oxidoreductase; NirK, copper-containing nitrite reductase; NirS, iron-containing nitrite reductase; Nor, nitric oxidase reductase. NirK is divided into two groups: one for nitrifier *nirK* transcripts most related to uncultured Thaumarchaeota thought to perform ammonia oxidation and nitrite-oxidizing bacteria, and the other in the denitrification pathway for all other *nirK* transcripts. Nar and Nxr are contained within the formate dehydrogenase protein fold family (SCOPe ID: c.81.1.1), NirK is contained within the multidomain cupredoxin protein fold family (SCOPe ID: b.6.1.3), Nor is contained within the ROO N-terminal domain-like protein fold family (SCOPe ID: d.157.1.3), and NirS is classified as SCOPe ID b.70.2.1 (C-terminus) and a.3.1.2 (N-terminus). Known nitrogen cycle metalloenzymes not yet in the SCOPe database (ammonia monooxygenase in nitrification and hydrazine synthase/reductase in anammox) are represented as colored arrows. Question mark indicates the unknown enzyme performing thaumarchaeotal hydroxylamine oxidation to nitrite.

Total Cu genes and transcripts ranged from 35 to 221 per 100,000 protein-coding genes and tended to decrease with depth and diminishing O_2_ (Figures 4A,B; Supplementary Table 5). With one exception, >60% of total Cu genes and transcripts at all depths and stations were associated with cytochrome c oxidase, the terminal reductase in aerobic respiration that catalyzes the transfer of electrons from reduced cytochrome to O_2_ (Figure 5). The proportional abundance of cytochrome c oxidase transcripts was positively correlated with O_2_ content (*p* > 0.001) and negatively correlated with depth (*p* = 0.02; Supplementary Table 6).

Genes encoding plastocyanin- and nitrosocyanin-like blue copper proteins, amine oxidases and Cu/Zn superoxide dismutases were negatively correlated with depth and positively correlated with O_2_ in OMZs (*p* < 0.05; Supplementary Table 6). Plastocyanin transcripts peaked at 11–14% of total Cu transcripts in the upper oxycline and upper OMZ, while Cu/Zn superoxide dismutase and amine oxidase comprised < 3% of total Cu transcripts (Figure 4B). Multi-copper oxidase (MCO) transcripts comprised 6–12% of total Cu transcripts in the upper OMZ and were dominated by *nirK* (see below). Other MCO and Cu genes were consistently < 1% of total sequences in all depths and stations (Supplementary Table 5).

Genes that code for Cu-containing metalloenzymes in the nitrogen cycle include nitrous oxide reductase (*nos*), the copper-containing form of nitrite reductase (*nirK*), and ammonia monooxygenase (*amo*, which is not yet included in the SCOP database, but was quantified by Ganesh et al. (2015) for ETNP station 6, Supplementary Table 5). Ammonia monooxygenase (*amoC*) transcripts with 95–100% similarity to *Nitrosopumilus* spp. contributed 48 and 42 transcripts per 100,000 protein-coding RNA sequences (~0.05% of total transcripts) at 30 and 85 m depth, respectively (Supplementary Table 5; Ganesh et al., 2015), suggesting that Amo may also contribute significantly to inventories of Cu proteins in the upper oxic zone.

Nitrous oxide reductase (*nos*) genes and transcripts were only detected in upper and core OMZs and comprised ~3% of total Cu sequences (Figures 4A,B, 5), consistent with previous findings for the ETNP (Ganesh et al., 2015) and ETSP (Dalsgaard et al., 2014). Moreover, Ganesh et al. (2014, 2015) showed that *nosZ* genes and transcripts are primarily associated with the >1.6 μm particle fraction, but comprised a small fraction of total protein-coding transcripts (0.005% at 85–100 m in the ETNP station 6; Supplementary Table 5). The low transcription of *nosZ* suggests that the Nos enzyme may be relatively stable in anoxic waters.

Copper-containing nitrite reductase (*nirK*) comprised up to 17% of Cu genes in upper OMZs and lower oxyclines vs. < 1% of Cu genes at BATS (Figure 4A). Because both nitrifying and denitrifying microbes may encode *nirK*, the relative abundance of *nirK* transcripts was separated into those two pathways in Figure 5. Transcripts of *nirK* reached 68% of total Cu RNA sequences in the upper OMZ at ETSP station 3 (Figure 4B) where O_2_ and NO${}_{2}^{-}$ concentrations were roughly equimolar in the low μM range (Figures 1A,B). The large spike in *nirK* in the upper ETSP OMZ originated from sequences with 95–100% identity to *nirK* from uncultured marine Thaumarchaeota, consistent with the dominance of thaumarchaeotal RNA sequences encoding Cu proteins at that depth (Figure 3D) and abundant thaumarchaeotal *nirK* sequences reported from the upper oxic zone and oxycline at station 6 in the ETNP (Supplementary Table 5; Ganesh et al., 2015). In the ETNP, *nirK* transcripts at 100–125 m depth had 75–90% sequence identity to *nirK* (WP_018047432.1) from *Nitrospina* sp. AB-629-B06, which itself had 66% identity similarity to *nirK* from *Nitrospina gracilis* (see discussion below).

### Iron genes and transcripts: relative abundance and functional composition

In OMZs, total gene sequences encoding Fe proteins (767–1888 per 100,000 protein-coding sequences; Figure 4C) were negatively correlated with O_2_ content (*p* < 0.01; Supplementary Table 6) and positively correlated with seawater Fe:Cu molar ratio (*p* < 0.001). At BATS, total Fe gene abundance decreased with depth (*p* < 0.001) and the most abundant Fe gene families were cytochrome c oxidase (8–11%) and catalase-peroxidase (6–11%), both of which also declined with depth (*p* < 0.001; Figure 4C; Supplementary Table 6). At all depths and stations, the most abundant class of Fe genes was “others” (37–63%), each of which represented < 4% of total Fe genes (Supplementary Table 5).

At least half of the DNA sequences encoding the two most abundant Fe-binding proteins appeared to be involved in NO${}_{3}^{-}$ respiration, the first step in the denitrification pathway that is encoded by dissimilatory nitrate reductase (*nar*) genes, with anammox genes not yet represented in the SCOP database likely also contributing significantly to the Fe gene inventory (see below). Genes in the formate dehydrogenase/DMSO reductase fold family (SCOPe ID: c.81.1.1) were significantly elevated in upper and core OMZs (*P* < 0.001; Figure 4C; Supplementary Table 6). Further, examination revealed that 43–47% of these genes were comprised of *narG*, which encodes the alpha subunit of the dissimilatory nitrate reductase protein, consistent with a previous report of abundant *narG* transcription in the ETNP core (Ganesh et al., 2015; Supplementary Table 5). Similarly, 45–50% of the sequences matching ferredoxin domains from multidomain proteins (SCOPe ID: d.58.1.5) had highest similarity to *narH* (dissimilatory nitrate reductase beta subunit) and were also negatively correlated with O_2_ (*p* < 0.001; Supplementary Table 6). Nitrite oxidoreductase (*nxrB*) genes also fell into the formate dehydrogenase/DMSO reductase fold family, and were most abundant in the upper OMZ (0.04% total protein-coding transcripts; Supplementary Table 5). Cytochrome nitrite reductase (*nirS*) grouped into the “others” category, and comprised < 1% of total gene sequences encoding Fe proteins (~0.03% of total transcripts) at 85 m in the ETNP at station 6 (Ganesh et al., 2015).

In contrast to Fe genes, total Fe transcripts (208–1124 per 100,000 protein-coding transcripts) displayed no statistically significant correlation with O_2_ or depth although individual families displayed significant correlations (Figure 4D; Supplementary Table 6). Photosystem II sequences comprising 64% of total Fe transcripts in the upper oxic zone were significantly negatively correlated with depth (*p* = 0.02; Figures 4D, 5; Supplementary Table 6) while Fe transcripts encoding cytochrome c oxidase were significantly positively correlated with O_2_ content (*p* = 0.03; Supplementary Table 6). Formate dehydrogenase transcripts with 60–90% similarity to *nar* genes from uncultured OP1 bacteria rose up to 34% total Fe transcripts in OMZ cores (Figures 4D, 5) as previously reported (Ganesh et al., 2014, 2015). It should be noted that formate dehydrogenase genes were present in upper oxic zones and transcribed in the upper oxycline at ETSP station 3 (Figure 4C), albeit at lower levels than in OMZs, and have been implicated in aerobic C1 metabolism by ubiquitous marine SAR11 Alphaproteobacteria (Sun et al., 2011). Iron-containing hydrazine synthase/oxidoreductase (*hzs/hzo*) involved in anammox metabolism were not included in this analysis because they have not yet been added to the SCOP database, but they were both abundant in the metatranscriptomes as analyzed by Ganesh et al. (2015), comprising up to 0.2% of total protein-coding transcripts in the OMZ core (Supplementary Table 5).

The relative abundance of genes and transcripts encoding Fe and Cu protein families were analyzed by partial CCA with ETNP environmental metadata (depth, temperature, O_2_, NO${}_{3}^{-}$, NO${}_{2}^{-}$, PO${}_{4}^{3-}$, dFe_T_, and dCu_T_; Supplementary Figure 2). Metagenomic and metatranscriptomic data from each OMZ zone type tended to cluster discretely. Oxic Fe and Cu genes were most strongly associated with increasing temperature and O_2_ content (Supplementary Figure 2). Interestingly, Fe transcripts from the upper OMZ showed a stronger relationship with increasing NO${}_{2}^{-}$ and Fe concentrations than Fe genes from the same samples, while Fe transcripts from the core OMZ displayed stronger correspondence with increasing NO${}_{3}^{-}$, PO${}_{4}^{3-}$ and depth (Supplementary Figure 2A). Transcripts encoding Cu proteins were most strongly associated with depth in upper OMZs (Supplementary Figure 2B).

### Comparison with genomes of dominant OMZ nitrogen-cycling microbes

Genes encoding Fe- and Cu-binding proteins were quantified using published genomes of marine prokaryotes (Supplementary Table 5). The *S. profunda* genome contained 261 genes encoding Fe-containing proteins (5.5% of 4756 predicted genes; Van de Vossenberg et al., 2013) including 35 copies of genes in the formate dehydrogenase/DMSO reductase fold family (SCOPe ID: c.81.1.1) and 13 Cu proteins (0.3% of predicted proteins). In contrast, the *N. maritimus* genome contained relatively more Cu genes including eight plastocyanins, six *nirK* genes encoding Cu-containing nitrite reductases, and two cytochrome c oxidases (0.8% of 1997 predicted proteins in genome; Walker et al., 2010) and only 20 Fe genes (1.0% of all predicted proteins). Nitrite-oxidizing *Nitrospinae* occupy O_2_ gradients in between those of strictly anaerobic Planctomycetes and aerobic-microaerophilic ammonia-oxidizing Thaumarchaeota (Füssel et al., 2011; Beman et al., 2013; Lücker et al., 2013), and accordingly possess intermediate numbers of genes encoding Fe and Cu proteins; the *N. gracilis* genome contained 46 Fe genes (1.5% of 3147 predicted genes; Lücker et al., 2013) and only 3 Cu genes (2 *nirK* genes and 1 cytochrome c oxidase; 0.1% of predicted genes). Below we discuss two specific examples of key metal requirements for OMZ nitrogen cycling in more detail: (1) Cu for marine ammonia-oxidizing Thaumarchaeota and (2) Fe for marine anammox Planctomycetes.

The obligate Cu requirement for the marine thaumarchaeote *Nitrosopumilus maritimus* SCM1 (Amin et al., 2013) is consistent with the dominance of marine Thaumarchaeota in aerobic to microaerophilic niches with Cu > Fe. Ammonia oxidation by pure cultures of *N. maritimus* is limited at log Cu^2+^ concentrations of −12.3 M (Amin et al., 2013), two orders of magnitude higher than Cu^2+^ in the ETNP waters we measured (−13.9 to −15 M; Supplementary Table 3), suggesting that rates of marine ammonia oxidation may be limited by inadequate Cu supplies, as supported by initial findings in a hypoxic fjord (Jacquot et al., 2014).

Strong peaks in *nirK* transcription suggest that the NirK enzyme may comprise a significant portion of microbial Cu utilization in OMZs. High levels of *nirK* expression and translation, respectively, have also been detected in coastal metatranscriptomes (Hollibaugh et al., 2011) and in a proteome of the marine ammonia-oxidizing thaumarchaeotal isolate “*Candidatus* Nitrosopelagicus brevis” (Santoro et al., 2015). Our data also suggest elevated transcription of *nirK* in nitrifying vs. denitrifying microbes (Figure 5) although the cellular purpose(s) of nitrifier NirK remains to be determined. NO accumulation has been observed following ammonium addition to cultures of the ammonia-oxidizing thaumarchaeote *Nitrosopumilus maritimus* SCM1, and was suggested to be an intermediate in ammonia oxidation and/or an electron shuttle to ammonia monooxygenase (Martens-Habbena et al., 2014). The function of *nirK* in nitrite-oxidizing *Nitrospina*, in which NO is not predicted to be an intermediate, is also unclear, but has been proposed to be involved in regulation of cellular redox state (Lücker et al., 2013 and refs therein). Nitrite accumulation in the secondary NO${}_{2}^{-}$ maximum may be at least partially a signature of Cu limitation in OMZs since pure cultures of *nirK*-containing denitrifying bacteria accumulate significantly more NO${}_{2}^{-}$ under Cu starvation than in Cu-replete conditions (Felgate et al., 2012). However, other environmental variables—perhaps most importantly the quantity and quality of organic carbon supply—also exert strong control on rates and products of marine denitrification (Ward et al., 2008; Babbin et al., 2014).

The very high abundance of genes encoding Fe-binding proteins in the anammox bacterium *Scalindua profunda* (5.5% of predicted genes) coupled with high transcription of *hzs/hzo* genes in the core of the ETNP OMZ (Supplementary Table 5) suggests that Fe might be an important micronutrient or energy source for planctomycetes mediating anammox (Strous et al., 2006; Klotz et al., 2008; Van de Vossenberg et al., 2013). A recent study showed that Fe and Cu were present at roughly equal intracellular levels in two anammox species (*Ca*. Jettenia caeni and *Ca*. Brocadia sinica), both of which possess MCO and *nirK* genes (Ali et al., 2014). Anammox bacteria have previously been shown to respire NO${}_{3}^{-}$, Fe^3+^ and Mn^4+^ with formate as the electron donor (Strous et al., 2006; Van De Vossenberg et al., 2008; Zhao et al., 2014), and planctomycete transcripts were present in the formate dehydrogenase sequences in the OMZs in this study, albeit as a minor component. Corresponding peaks of Fe^2+^ and anammox rates in the ETSP suggest the intriguing possibility that marine anammox bacteria may reduce Fe^3+^ in OMZs (Kondo and Moffett, 2015), but the importance of this process in the marine Fe cycle remains to be investigated.

## Conclusions

This study revealed significant correlations between marine O_2_, Fe and Cu concentrations, and microbial gene and transcript inventories at the community level for selected metalloenzymes in meta-omic datasets from natural marine ecosystems. Most significantly, it showed for the first time significant inverse relationships between seawater molar Fe:Cu ratio and O_2_ concentration, and microbial Fe gene abundance and O_2_ content, as well as a significant positive correlation between microbial cytochrome c oxidase transcripts and O_2_ content. While we cannot rule out the possibility that minor changes in mRNA transcript abundances occurred during sampling in response to shifting environmental (e.g., O_2_) conditions (Feike et al., 2012; Moran et al., 2013; Stewart, 2013), we consider such changes to be relatively minimal given the observed depth-specific structuring of the transcript pool, with a clear shift from aerobic to anaerobic metabolisms with depth in the OMZ, and given our attempts to minimize time between sample recovery and RNA preservation (~30 min).

While meta-omic signatures of micro-nutrient limitation in marine phytoplankton have been the focus of numerous recent investigations (e.g., Bertrand et al., 2012; Chappell et al., 2012; Hopkinson and Barbeau, 2012; Moore et al., 2013; Saito et al., 2014), trace metal requirements for marine anaerobic microbes remain largely uncharacterized. The data reported here suggest the importance of Fe and Cu as enzyme cofactors for anaerobic and aerobic/microaerophilic microbial dissimilatory N metabolisms such as denitrification/anammox and ammonia oxidation, respectively. Inclusion of Cu-containing ammonia monooxygenase (*amo*) and Fe-containing hydrazine synthase/oxidoreductase (*hzs/hzo*) sequences in future versions of the SCOP database would enable further tests of these hypotheses.

Comparative genomic analyses of cultivated microbes have revealed that the majority of microbial genomes from aerobes contain at least one Cu-binding protein, whereas the majority of genomes from strict anaerobes do not (Ridge et al., 2008). Since some Fe and Cu proteins can perform similar metabolic functions (e.g., cytochromes and plastocyanins in electron transfer), it is possible that long-term evolutionary pressure favored a relatively greater use of Cu in aerobic metabolisms in oxic seawater with Cu > Fe and, conversely, greater use of Fe in anaerobic metabolisms in anoxic seawater with Fe > Cu. Indeed, it has been suggested that the abundance of Fe proteins required for anammox reflects the evolution of this pathway prior to the Great Oxidation Event, in contrast to Cu proteins involved in aerobic ammonia oxidation that likely emerged after the rise of atmospheric O_2_ (Klotz and Stein, 2008).

Looking to the future, changes in trace metal bioavailability as a function of seawater O_2_ content will likely continue to exert evolutionary pressure by shaping functional gene inventories of marine microbes. Expansion of OMZs could lead to higher demands for Fe and Cu for metalloenzyme activity. Higher rates of water column Fe^3+^ reduction might support this activity, although inputs of metals from terrestrial, sediment or hydrothermal sources would ultimately limit their availability. Further examinations of relationships between micronutrient availability and gene abundance/distribution may have predictive value in informing models that use chemical distributions to predict microbial metabolic processes.

### Conflict of interest statement

The authors declare that the research was conducted in the absence of any commercial or financial relationships that could be construed as a potential conflict of interest.

## Abbreviations

BATS: Bermuda Atlantic Time-series Station
DMSO: dimethyl sulfoxide
ETNP: Eastern Tropical North Pacific
ETSP: Eastern Tropical South Pacific
OMZ: oxygen minimum zone
SCOP: Structural Classification of Proteins
MCO: multi-copper oxidase
dFeT: total dissolved iron
dCuT: total dissolved copper.
