# Corrigendum: Meta-omic signatures of microbial metal and nitrogen cycling in marine oxygen minimum zones

**DOI:** 10.3389/fmicb.2020.619943

**Published:** 2020-12-23

**Authors:** Jennifer B. Glass, Cecilia B. Kretz, Sangita Ganesh, Piyush Ranjan, Sherry L. Seston, Kristen N. Buck, William M. Landing, Peter L. Morton, James W. Moffett, Stephen J. Giovannoni, Kevin L. Vergin, Frank J. Stewart

**Affiliations:** ^1^School of Earth and Atmospheric Sciences, Georgia Institute of Technology, Atlanta, GA, United States; ^2^School of Biology, Georgia Institute of Technology, Atlanta, GA, United States; ^3^Department of Biology, Alverno College, Milwaukee, WI, United States; ^4^College of Marine Science, University of South Florida, St. Petersburg, FL, United States; ^5^Department of Earth, Ocean and Atmospheric Sciences, Florida State University, Tallahassee, FL, United States; ^6^Department of Biological Sciences, University of Southern California, Los Angeles, CA, United States; ^7^Department of Microbiology, Oregon State University, Corvallis, OR, United States

**Keywords:** oxygen minimum zones, metalloenzymes, iron, copper, denitrification, anammox, metagenomes, metatranscriptomes

In the original article, there was a mistake in Figure 1, panels B and C, as published. The nitrite (Figure 1B) and nitrate (Figure 1C) depth profiles for ETNP Stn 2 and ETSP Stn 3 were incorrectly plotted. The corrected Figure 1 appears below.

**Figure 1:**
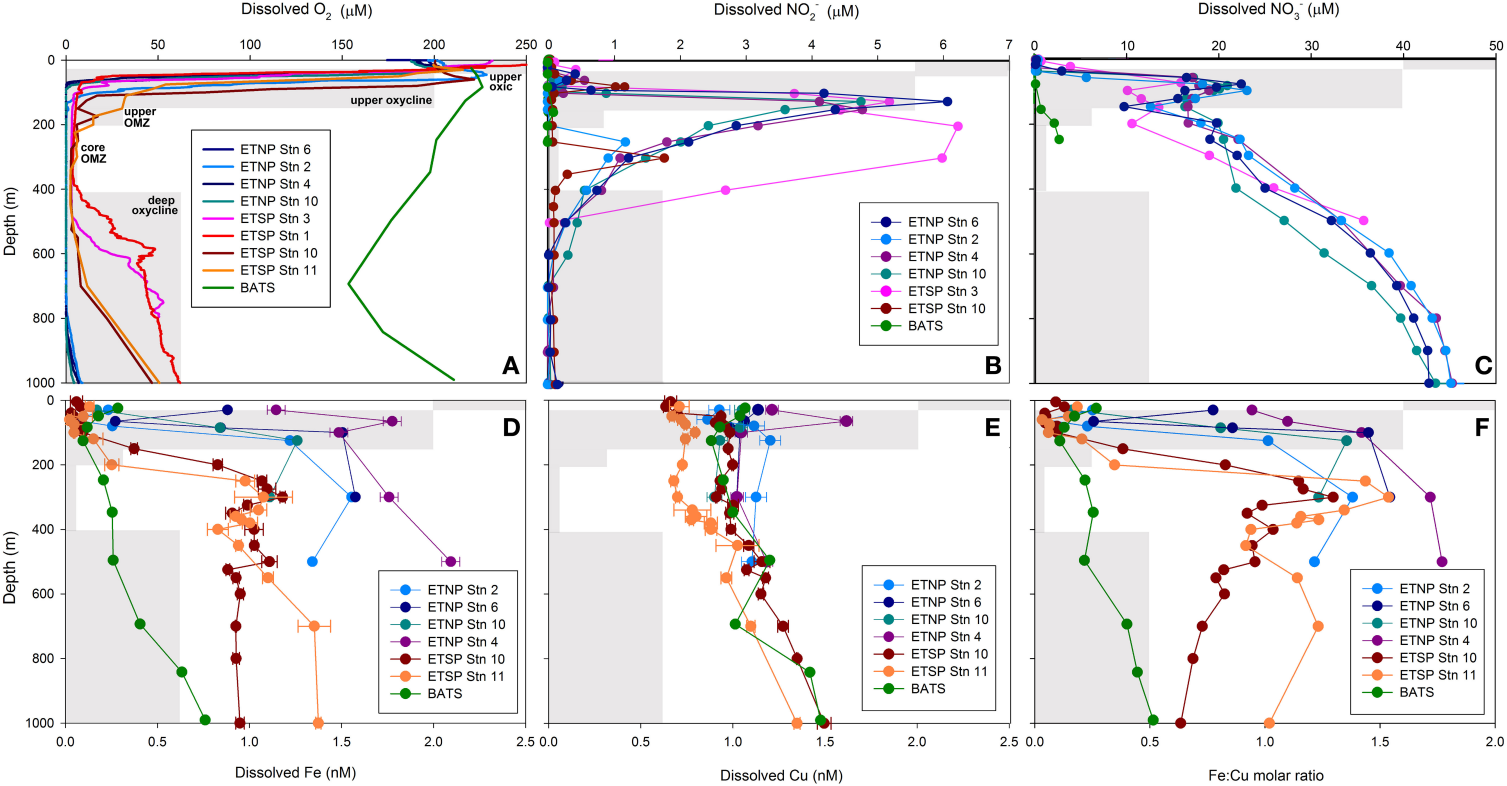
Depth profiles of dissolved **(A)** O_2_, **(B)** ${\text{NO}}_{2}^{-}$, **(C)** ${\text{NO}}_{3}^{-}$, **(D)** Fe, **(E)** Cu and **(F)** Fe:Cu molar ratios for stations 2, 4, 6 and 10 in the ETNP, stations 1 (BIG RAPA) and 3 (MOOMZ), 10 and 11 in the ETSP, and BATS in the Sargasso Sea, North Atlantic Ocean (see **Supplementary Figure 1** for station maps). Gray boxes depict oxygen and depth ranges for each zone and their labels are shown in **(A)**.

The authors apologize for this error and state that this does not change the scientific conclusions of the article in any way. The original article has been updated.

