# Supplementary material for: Meta-omic signatures of microbial metal and nitrogen cycling in marine oxygen minimum zones
Source: Front Microbiol. 2015 Sep 28;6:998. doi: 10.3389/fmicb.2015.00998 (PMC4585252; doi:10.3389/fmicb.2015.00998)
Supplement: Supplementary file 1 [file DataSheet1.PDF]

Supplementary Material

Meta-omic signatures of microbial metal and nitrogen cycling in marine oxygen minimum zones

Authors: Jennifer B. Glass[a,b*], Cecilia Batmalle Kretz[a], Sangita Ganesh[b], Piyush Ranjan[b], Sherry L. Seston[c], Kristen N. Buck[d], William M. Landing[e], Peter L. Morton[e], James W. Moffett[f], Stephen J. Giovannoni[g], Kevin L. Vergin[g], Frank J. Stewart[a,b]

***Correspondence:** Corresponding Author: jennifer.glass@eas.gatech.edu

1. Supplementary Figures and Tables

1.1. Supplementary Figures

1.1.1. Supplementary Figure 1

1.1.2. Supplementary Figure 2

1.2. Supplementary Tables

1.2.1. Supplementary Table 1

1.2.2. Supplementary Table 2

1.2.3. Supplementary Table 3

1.2.4. Supplementary Table 4

1.2.5. Supplementary Table 5 (appended Excel spreadsheet)

1.2.6. Supplementary Table 6

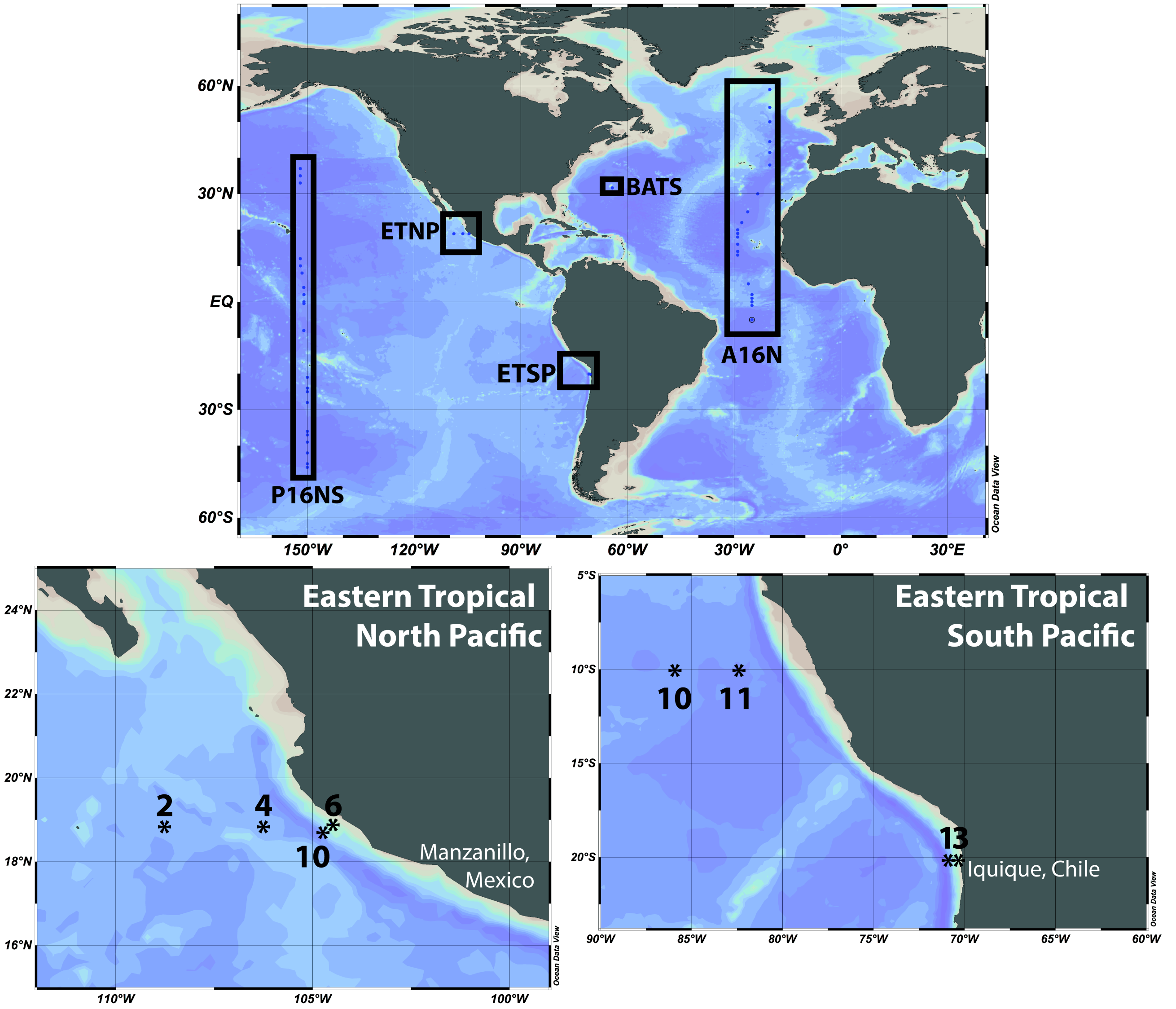

Supplementary Figure 1. Maps showing locations of sampling sites. Stations include Eastern Tropical North Pacific (ETNP) stations 2, 4, 6 and 10 (sampled in 2013), Eastern Tropical South Pacific (ETSP) stations 1 (BIGRAPA, sampled in 2010), 3 (MOOMZ, sampled in 2008), 10 and 11 (sampled in 2010), Bermuda Atlantic Time-series Station (BATS; sampled in 2002), and Climate and Ocean: Variability, Predictability and Change (CLIVAR) transects A16N (sampled in 2003) in the Atlantic Ocean and P16N (sampled in 2005) and P16N (sampled in 2006) in the Pacific Ocean.

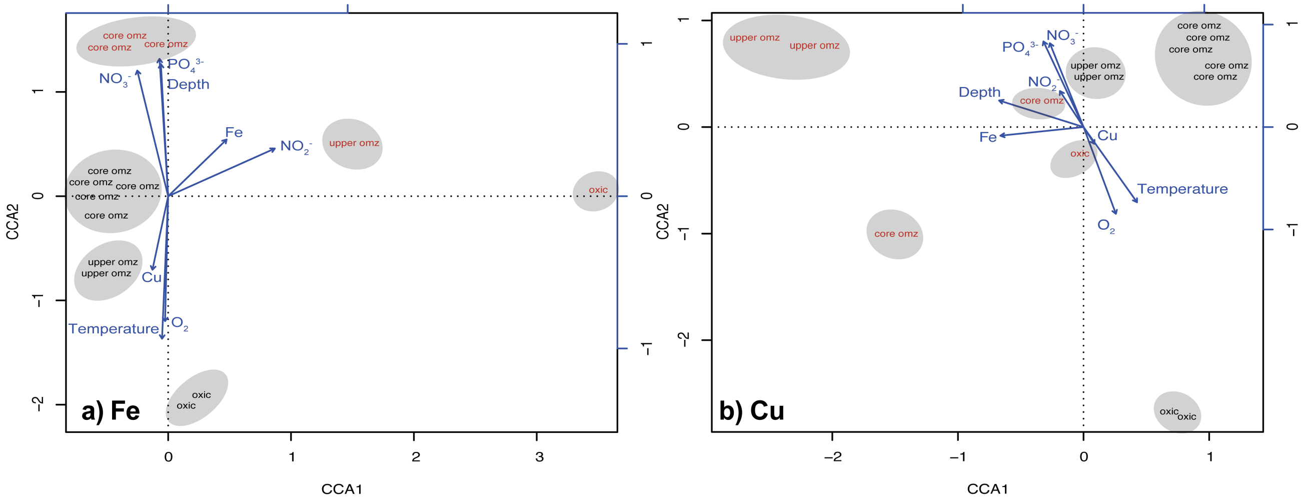

Supplementary Figure 2. Partial canonical correspondence analysis for (a) Fe and (b) Cu gene and transcript abundances based on depth, temperature, dissolved O_2, NO_3^-, NO_2^-, PO_4^{3-}, Fe and Cu concentration. Data are presented from oxic, upper OMZ and core OMZ samples collected on ETNP cruise NH-1315 for metagenomes (black text) and metatranscriptomes (red text). Total constrained inertia was 0.8 for Fe gene/transcript abundances and 0.2 for Cu gene/transcript abundances and 100% of the variance was explained by the environmental variables.

Supplementary Table 1. Sequencing statistics and accession numbers for metagenomes from ETNP stations 6 and 10 and BATS.

Sample	Total reads	Mean read length (bp)	Total protein-coding reads matching NCBI-nr with bit >50	*NCBI BioSample (for ETNP) or iMicrobe (for BATS) accession number*
ETNP Station 6				
30 m	1,689,674	249	1,223,450	SAMN02905556
85 m	2,315,878	234	1,654,940	SAMN02905557
100 m	1,525,747	239	1,055,130	SAMN02905558
125 m	1,042,562	242	721,275	SAMN02905559
300 m	4,591,750	234	3,161,230	SAMN02905560
ETNP Station 10				
30 m	374,345	276	272,252	SAMN02905561
80 m	2,618,866	281	2,020,380	SAMN02905562
125 m	2,679,134	283	2,009,600	SAMN02905563
300 m	2,310,714	284	1,791,490	SAMN02905564
BATS				
1m	635,327	216	276,944	BATS_SMPL_BATS-167-0
40m	577,748	210	187,088	BATS_SMPL_BATS-167-40
80m	604,433	218	235,986	BATS_SMPL_BATS-167-80
120m	535,943	220	203,643	BATS_SMPL_BATS-167-120
160m	331,401	222	144,819	BATS_SMPL_BATS-167-160
200m	658,537	225	292,492	BATS_SMPL_BATS-167-200
250m	562,825	220	193,318	BATS_SMPL_BATS-167-250

Supplementary Table 2. Total dissolved Fe (dFe_T) concentrations, labile inorganic Fe complexes ([Fe']), Fe-L_1 ligand concentrations and conditional stability constants ($logK_{Fe-L1}$) through the ETNP OMZ. Parentheses represent standard deviations (n=3). White boxes indicate upper oxic samples (O_2 > 200 μM), light gray boxes indicate oxycline samples (O_2 = 10-200 μM) and dark gray boxes indicate upper and core OMZ samples (O_2 < 10 μM).

Depth (m)	Stn 2				Stn 4				Stn 6				Stn 10			
	dFe_T (nM)	[Fe'] (pM)	L_1 (nM)	LogK	dFe_T (nM)	[Fe'] (pM)	L_1 (nM)	LogK	dFe_T (nM)	[Fe'] (pM)	L_1 (nM)	LogK	dFe_T (nM)	[Fe'] (pM)	L_1 (nM)	LogK
30	0.23 (0.01)	0.07	1.10 (0.01)	12.58 (0.01)	1.14 (0.05)	0.15	2.09 (0.05)	12.87 (0.12)	0.88 (0.01)	0.16	1.70 (0.05)	12.79 (0.11)	0.17 (0.01)	0.03	2.29 (0.06)	12.78 (0.15)
60	0.04 (0.01)	0.02	1.07 (0.01)	12.35 (0.02)												
65					1.78 (0.05)				0.27 (0.01)							
80	0.25 (0.01)	0.07	1.52 (0.04)	12.44 (0.05)												
85									0.84 (0.01)				0.84 (0.01)			
100					1.49 (0.05)	0.43	2.56 (0.03)	12.47 (0.05)	1.50 (0.01)	0.36	2.40 (0.04)	12.60 (0.05)				
125	1.22 (0.01)	0.26	2.26 (0.02)	12.60 (0.02)									1.26 (0.01)			
300	1.55 (0.01)	0.31	2.37 (0.03)	12.73 (0.05)	1.76 (0.05)	0.45	2.84 (0.08)	12.53 (0.17)	1.58 (0.01)	0.39	2.67 (0.07)	12.52 (0.11)	1.11 (0.01)	0.43	2.73 (0.05)	12.52 (0.07)
500	1.34 (0.01)	0.28	2.25 (0.02)	12.66 (0.03)	2.09 (0.05)	0.60	3.03 (0.06)	12.54 (0.08)								

Supplementary Table 3. Total dissolved Cu (dCu_T) concentrations, log Cu^{2+} concentrations, Cu-L_1 ligand concentrations and conditional stability constants ($logK_{Cu\text{-}L1}$) through the ETNP OMZ. Parentheses represent standard deviations (n=3). White boxes indicate upper oxic samples (O_2 > 200 μM), light gray boxes indicate oxycline samples (O_2 = 10-200 μM) and dark gray boxes indicate upper and core OMZ samples (O_2 < 10 μM).

Depth (m)	Stn 2				Stn 4				Stn 6				Stn 10			
	dCu_T (nM)	Log Cu^{2+} (M)	L_1 (nM)	LogK	dCu_T (nM)	Log Cu^{2+} (M)	L_1 (nM)	LogK	dCu_T (nM)	Log Cu^{2+} (M)	L_1 (nM)	LogK	dCu_T (nM)	Log Cu^{2+} (M)	L_1 (nM)	LogK
30	0.93 (0.06)	-14.26	2.49 (0.07)	13.98 (0.08)	1.21 (0.03)	-14.18	2.74 (0.38)	13.97 (0.16)	1.14 (0.01)	-14.79	3.35 (0.01)	14.45 (0.01)	1.05 (0.04)	-14.81	5.84 (0.23)	14.12 (0.08)
60	0.86 (0.06)	-14.94	3.47 (0.05)	14.42 (0.06)												
65					1.62 (0.03)				1.06 (0.01)							
80	1.12 (0.06)	-14.77	4.01 (0.13)	14.33 (0.18)												
85									0.98 (0.01)				1.04 (0.04)			
100					1.05 (0.03)	-14.39	3.30 (0.17)	14.00 (0.06)	1.04 (0.01)	-14.61	2.87 (0.09)	14.34 (0.15)				
125	1.20 (0.06)	-15.00	3.06 (0.20)	14.81 (0.58)									0.93 (0.04)			
300	1.13 (0.01)	-13.92	2.32 (0.06)	13.82 (0.06)	1.02 (0.03)	-14.40	3.02 (0.42)	14.08 (0.43)	1.02 (0.01)	-14.85	1.98 (0.01)	14.78 (0.01)	0.90 (0.04)	-14.42	3.64 (0.20)	13.94 (0.11)
500	1.10 (0.06)	-14.88	2.42 (0.09)	14.80 (0.29)	1.18 (0.03)	-14.24	3.08 (0.24)	13.98 (0.11)								

Supplementary Table 4. Domain level characterization (in % of total protein-coding genes, for Bacteria (B), Archaea (A), and Eukaryotes (E)) for bulk protein-coding genes/transcripts ("Bulk"), genes/transcripts encoding Cu-containing proteins ("Cu"), and genes/transcripts encoding Fe-containing proteins ("Fe").

		**Bulk-MG**			**Cu-MG**			**Fe-MG**			**Bulk-MT**			**Cu-MT**			**Fe-MT**		
**Zone**	**Station: depth (m)**	**B**	**A**	**E**	**B**	**A**	**E**	**B**	**A**	**E**	**B**	**A**	**E**	**B**	**A**	**E**	**B**	**A**	**E**
oxic	**BATS-167: 1m**	96	0	4	93	0	7	98	0	3									
oxic	**BATS-167: 40m**	94	0	5	95	0	5	97	0	3									
oxic	**BATS-167: 80m**	94	1	4	92	2	5	97	1	2									
oxic	**BATS-167: 120m**	92	3	5	96	4	0	96	1	3									
oxic	**BATS-167: 160m**	87	8	5	90	10	0	94	4	3									
oxic	**BATS-167: 200m**	86	9	5	83	9	7	91	5	3									
oxic	**BATS-167: 250m**	88	7	4	88	6	7	93	5	2									
upper oxic	**ETSP-03: 15m**	89	5	5	89	3	6	93	2	4									
upper oxic	**ETNP-06: 30m**	86	12	1	85	12	2	92	6	1	75	7	19	77	8	14	84	5	11
upper oxic	**ETNP-10: 30m**	87	7	4	90	6	4	92	5	3									
upper oxycline	**ETSP-03: 50m**	85	12	2	82	15	3	90	8	2	74	15	11	63	19	18	85	6	8
upper oxycline	**ETSP-03: 65m**	81	17	1	81	19	0	87	11	2									
upper oxycline	**ETSP-03: 85m**	82	16	1	82	18	0	88	10	1	69	20	11	44	44	12	85	11	4
upper OMZ	**ETSP-01: 70m**	90	7	1	87	11	3	94	4	1									
upper OMZ	**ETNP-10: 80m**	84	15	1	78	20	2	87	11	1									
upper OMZ	**ETNP-06: 85m**	89	10	0	85	12	2	91	7	1	86	4	9	76	11	13	90	4	6
upper OMZ	**ETNP-06: 100m**	94	4	1	92	4	3	95	3	1	94	2	4	85	0	15	95	2	3
upper OMZ	**ETSP-03: 110m**	94	3	1	91	3	6	96	2	1	83	15	2	30	67	3	87	11	2
upper OMZ	**ETSP-01: 110m**	90	8	1	88	12	0	95	4	1									
upper OMZ	**ETNP-10: 125m**	94	4	1	91	3	6	95	4	1									
upper OMZ	**ETNP-06: 125m**	94	3	1	91	3	5	95	3	1	93	3	4	100	0	0	95	2	3
core OMZ	**ETSP-01: 200m**	96	2	1	95	0	5	98	1	1									
core OMZ	**ETSP-03: 200m**	84	15	1	96	2	2	98	1	1	96	2	2	100	0	0	100	0	0
core OMZ	**ETNP-06: 300m**	95	3	1	91	1	8	95	3	1	94	3	3	100	0	0	100	0	0
core OMZ	**ETNP-10: 300m**	96	2	1	90	1	9	97	2	1									
deep oxycline	**ETSP-03: 500m**	83	16	1	80	20	0	90	9	1									
deep oxycline	**ETSP-03: 800m**	77	22	1	71	30	0	87	11	2									
deep oxycline	**ETSP-01: 1000m**	75	22	1	69	32	0	87	11	1									

Supplementary Table 5. Fe and Cu-binding SCOPe fold families used in bioinformatics analyses from Dupont et al. (2006, 2010), number of genes encoding each fold family in genomes of ammonia-oxidizing thaumarchaeote *Nitrosopumilus maritimus*, nitrite-oxidizing bacterium *Nitrospina gracilis* and anammox planctomycete *Scalindua profunda,* and number of transcripts encoding each fold family in metagenomes from ETNP station 6 and ESTP station 3. "Other" fold families each represented <4% of total genes encoding Fe or Cu-binding proteins in OMZ metagenomes. Red and green text indicate fold families used to construct Figure 5.

[See Appended Excel Spreadsheet]

Supplementary Table 6. Results of Spearman's rank correlations for individual copper (Cu) and iron (Fe) binding protein families and their total relative abundances. "MG" signifies "metagenomes" and "MT" signifies "metatransriptomes". For pairs of variables with positive correlation coefficients (ρ), sequence abundance increases with increasing O_2 or depth. For pairs of variables with negative correlation coefficients (ρ), sequence abundance decreases with increasing O_2 or depth. Significant correlations are indicated by asterixed, with * indicates p values between 0.01-0.05, **indicates p values between 0.001-0.01, and *** indicates p values <0.001. "ND" means no reads for statistical calculations.

Protein Families		OMZs-MG				BATS-MG				OMZs-MT			
		O_2		Depth		O_2		Depth		O_2		Depth	
		ρ	p	ρ	p	ρ	p	ρ	p	ρ	p	ρ	p
Cu	Cytochrome c oxidase	-0.2	0.4	-0.4	0.06	0.0	1.0	-0.9	0.000002 ***	0.9	0.0000002 ***	-0.7	0.02 *
	Nitrite reductase *(nirK)*	-0.2	0.3	-0.2	0.3	0.1	0.8	0.7	0.1	-0.1	0.9	0.3	0.4
	Plastocyanin/nitrosocyanin	0.8	0.000002 ***	-0.7	0.001 **	0.6	0.1	-0.6	0.1	0.4	0.2	-0.4	0.2
	Amine oxidases	0.7	0.0009 ***	-0.5	0.02 *	-0.8	0.03 *	0.1	0.7	0.4	0.3	-0.1	0.7
	Cu,Zn superoxide dismutase	0.5	0.03 *	-0.8	0.0000002 ***	-0.1	0.8	-0.3	0.5	0.2	0.6	-0.3	0.4
	Multicopper oxidase	0.01	1.0	-0.3	0.2	0.2	0.6	-0.4	0.3	ND	ND	ND	ND
	Nitrous oxide reductase (*nosZ*)	-0.4	0.09	-0.2	0.4	-0.1	0.8	0.8	0.04 *	0.0	0.9	0.3	0.5
	Others	0.3	0.3	-0.2	0.4	-0.4	0.3	-0.3	0.5	0.2	0.6	-0.3	0.4
	Total	-0.02	0.9	-0.6	0.003 **	0.0	1.0	-0.9	0.0000002 ***	0.8	0.004 **	-0.4	0.2
Fe	Cytochrome c oxidase	-0.2	0.3	-0.4	0.1	0.1	0.8	-1.0	0.00000002 ***	0.7	0.03 *	-0.3	0.4
	Formate dehydrogenase	-0.8	0.000002 ***	0.4	0.1	-0.7	0.05	0.5	0.2	-0.4	0.3	0.5	0.1
	Ferredoxin domains	-0.8	0.0000002 ***	0.5	0.03 *	-0.7	0.06	-0.4	0.4	-0.3	0.4	0.5	0.2
	2Fe-2S ferredoxin domains	-0.6	0.006 **	0.2	0.3	-0.6	0.1	0.6	0.1	-0.2	0.7	0.4	0.2
	CO dehydrogenase	-0.8	0.000002 ***	0.6	0.01 **	-0.4	0.4	0.7	0.1	-0.1	0.8	0.5	0.1
	Cytochrome b	-0.3	0.2	-0.1	0.6	0.5	0.3	-0.7	0.1	0.6	0.1	-0.3	0.5
	Photosystem II	0.4	0.05	-0.8	0.0000002 ***	0.5	0.2	-0.4	0.3	0.3	0.4	-0.7	0.02 *
	Catalase/peroxidase	0.8	0.000002 ***	-0.5	0.03 *	-0.1	0.8	-1.0	0.000002 ***	0.3	0.4	-0.5	0.2
	Ribonucleotide reductase	0.7	0.0005 ***	-0.6	0.002 **	0.2	0.6	-0.4	0.4	0.6	0.06	-0.2	0.5
	Others	-0.4	0.07	0.1	0.5	0.04	0.9	-0.3	0.5	0.4	0.3	0.1	0.8
	Total	-0.6	0.003 **	0.1	0.6	0.04	0.9	-0.8	0.04 *	0.5	0.2	-0.1	0.7
